# Evaluation of the Possible Correlation Between Dental Occlusion and Craniomandibular Disorders by Means of Teethan^®^ Electromyography: Clinical-Observational Study on 20 Patients

**DOI:** 10.3390/jcm14155508

**Published:** 2025-08-05

**Authors:** Vito Crincoli, Alessio Danilo Inchingolo, Grazia Marinelli, Rosalba Lagioia, Paola Bassi, Claudia Ciocia, Francesca Calò, Roberta Deodato, Giulia Marsella, Francesco Inchingolo, Andrea Palermo, Mario Dioguardi, Angela Pia Cazzolla, Maria Severa Di Comite, Maria Grazia Piancino, Angelo Michele Inchingolo, Gianna Dipalma

**Affiliations:** 1 Interdisciplinary Department of Medicine, School of Medicine, University of Bari “Aldo Moro”, 70124 Bari, Italy; vito.crincoli@uniba.it (V.C.); rosalba.lagioia@uniba.it (R.L.); paola.bassi@uniba.it (P.B.); claudia.ciocia@uniba.it (C.C.); francesca.calo@uniba.it (F.C.); robertadeo97@gmail.com (R.D.); francesco.inchingolo@uniba.it (F.I.); angelomichele.inchingolo@uniba.it (A.M.I.); gianna.dipalma@uniba.it (G.D.); 2 Private Practice Via Salvatore Matarrese 2/13, 70124 Bari, Italy; giulymarsella@gmail.com; 3 Department of Experimental Medicine, University of Salento, 73100 Lecce, Italy; andrea.palermo@unisalento.it; 4 Department of Clinical and Experimental Medicine, University of Foggia, 71122 Foggia, Italy; mario.dioguardi@unifg.it (M.D.); angelapia.cazzolla@unifg.it (A.P.C.); 5 Department of Translational Medicine and Neuroscience, University of Bari, 70124 Bari, Italy; mariasevera.dicomite@uniba.it; 6 Department of Surgical Sciences, Dental School, University of Turin, 10126 Turin, Italy; mariagrazia.piancino@unito.it

**Keywords:** temporomandibular joint, electromyography, Teethan^®^, masticatory muscles, muscle pain, diagnostic test, muscle activity, occlusal asymmetry, mandibular torsion, bruxism

## Abstract

**Background:** Temporomandibular disorders are a generic term referred to clinical conditions involving the jaw muscles and temporomandibular joint with multifactorial pattern and genetic background. The aim of this observational study was to investigate the correlation between craniomandibular disorders and the presence of occlusal alterations. A clinical evaluation of the occlusal and articular status of the patients was carried out, integrating the latter with the electromyographic recording the activity of the masseter and temporalis muscles. **Methods:** A clinical observational study on 20 adults assessed temporomandibular disorders using DC/TMD criteria, anamnesis, clinical exams, occlusal and electromyographic analyses. Occlusion was evaluated morphologically and functionally. Electromyography tested static/dynamic muscle activity. Data were statistically analyzed using *t*-tests and Pearson correlation (*p* < 0.05). **Results:** Electromyographic analysis revealed significant differences between subjects with and without visual correction, suggesting that visual input influences masticatory muscle activity. Correlations emerged between occlusal asymmetries and neuromuscular parameters. These findings highlight clinical implications for mandibular function, muscle symmetry, and the potential for therapeutic rebalancing through targeted interventions. **Conclusions:** The study demonstrates a significant correlation between visual–motor integration and masticatory muscle efficiency. It emphasizes lateralized neuromuscular activation’s influence on occlusal contact distribution. Moreover, it identifies mandibular torsion–endfeel inverse correlation as a potential diagnostic marker for craniomandibular dysfunctions via surface electromyography.

## 1. Introduction

Temporomandibular disorders (TMDs) are considered a distinct subgroup of musculoskeletal and rheumatoid disorders involving the temporomandibular joint (TMJ) and the jaw muscles [1,2,3,4]. They represent the most important cause of orofacial pain of non-dental origin, with up to 93% of the general population showing at least one TMD symptom or sign [5,6,7,8,9]. TMDs include pain, headaches located in temporal area, masticatory muscle fatigue and limited mandibular movement [10,11,12,13]. These altered functions can lead to difficulties in eating, drinking and swallowing, causing a TMD-related oral stage dysphagia (OD) and weight loss [14,15,16,17].

The pattern of TMDs is multifactorial, and among the main associated etiological factors are the following:

- Traumas (macro traumas such as a car accident, a fight or a fall; microtraumas such as titillophagia and onychophagia);
- Occlusal factors (second/third classes, unilateral crossbite, open bite);
- Hormonal factors (possible role of estrogens in the excessive laxity of the ligaments);
- Psychological causes (increased emotional tension and excess stress);
- Wakeful or sleeping bruxism;
- Genetic background (XX) [18].

From an epidemiological point of view, there is a clear prevalence of TMDs in females, with an F:M ratio of 7:1, probably linked to greater laxity of the ligament structures, greater sensitivity to emotional stress and a possible hormonal role. In the International Headache Society (IHS) 1988 “Classification of Cephalalgic Disorders of Cranial Neuralgia and Facial Pain,” orofacial pain and TMJ disorders fall under section 11 “headaches or facial pain associated with disorders of the skull, neck, eyes, ears, nose and sinuses, teeth, mouth, or other facial or cranial structures [19,20,21,22,23].” The AAOP (American Academy of Orofacial Pain) two years later suggested the addition of a subgroup covering masticatory muscle disorders [24,25,26,27,28]. With the aim of guiding the clinician in the diagnostic flow, in 1992, Dworkin proposed the Research Diagnostic Criteria for Temporomandibular Disorder (DC/TMD), which provides a dual-axis diagnostic system for craniomandibular disorders [29,30,31,32]. Axis I distinguishes muscular, joint and skeletal pathologies on a clinical basis; Axis II refers to the emotional and psychological nature of TMDs often associated with disorders of the anxious-depressive component, mood disorders and hypochondriasis [33,34,35,36].

Subsequently, the database INfORM (International Network for Orofacial Pain and Related Disorders Methodology) proved to be a useful tool in the development and dissemination of the Diagnostic Criteria for Temporomandibular Disorders (DC/TMD) (doi: 10.11607/jop.1151).

The objective of this study is to evaluate the possible correlation between craniomandibular disorders and occlusal alterations through a clinical evaluation of the occlusal and articular status of the subjects, integrating the latter with the electromyographic recording of the activity of the masseter (MM) and temporalis muscles [37,38,39,40,41]. Electromyography (EMG) refers to a functional diagnostic technique for recording and analysing the myoelectric signal, i.e., the electrical bio-potential that concerns muscle activity during contraction [42,43,44,45,46].

In particular, in the field of dentistry, craniomandibular electromyography and kinesiography, promoted by Jankelson, provides objective diagnostic measurements, while TENS, i.e., transcutaneous neural electrical stimulation, allows the achievement of a correct registration of the muscular centric relationship [47,48,49,50,51].

Depending on how the signal is recorded, a surface electromyography (sEMG) or “needle” electromyography (also called electroneurography, ENG) can be used. sEMG involves the sampling of the signal via electrodes placed on the skin (superficial electrodes), while needle EMG involves the use of subcutaneous needles placed in direct contact with the muscle of interest (the needle will be longer the deeper the muscle is) [52,53,54,55]. The recorded potentials will therefore highlight a group of motor units (nerves) and their conduction speed with the surface electrodes, while with the needle electrodes, it will be possible to analyze a single motor unit (the EMG allows you to “look” directly into the muscle) [55,56,57,58].

Needle EMG is an invasive neurophysiological diagnostic technique that measures the electrical activity of muscles directly within them.

Needle EMG offers a number of crucial diagnostic advantages, particularly in the evaluation of neuromuscular disorders:

- It allows analysis of the shape, amplitude, duration and frequency of action potentials of individual motor units, providing detailed information on the integrity of the peripheral nerve and muscle.
- It can detect abnormal electrical activity at rest (such as fibrillations or positive waves), which is indicative of muscle denervation (nerve damage).
- It evaluates how motor units are activated during voluntary contraction, which is useful for distinguishing between neurogenic (nerve damage) and myogenic (muscle damage) disorders.
- It is essential for distinguishing between a wide range of neuromuscular disorders, including peripheral nerve damage (carpal tunnel syndrome, diabetic neuropathies, radiculopathies from herniated discs, traumatic nerve injuries); myopathies (muscular dystrophies, myositis); diseases that alter nerve-to-muscle signal transmission (myasthenia gravis); diseases that affect motor neurons in the brain and spinal cord (amyotrophic lateral sclerosis).

It often allows precise localization of the level of damage (e.g., nerve root, plexus, peripheral nerve, neuromuscular junction, or muscle itself). It can be performed on any muscle accessible with the needle, including deep muscles that would otherwise be inaccessible with surface electrodes. This allows for a targeted assessment based on the patient’s symptoms. It is a highly sensitive test for detecting even minimal or early alterations in neuromuscular activity, sometimes before obvious clinical symptoms appear.

Needle EMG also has some significant limitations:

- Invasiveness and Patient Discomfort
- Pain/Discomfort: Inserting the needle into the muscle can be painful or uncomfortable for the patient. Although the needles are thin and disposable, pain tolerance varies from person to person.
- Anxiety: Many patients experience anxiety at the thought of needle insertion, which can make the examination more difficult.
- Bruising/Bleeding: Minor bruising or slight bleeding may occur at the puncture sites, especially in patients with coagulation disorders or who are on anticoagulant therapy.
- Risk of Complications (Rare): Infections, although rare with sterile, disposable needles, there is a minimal risk of infection at the puncture site: pneumothorax—extremely rare, but theoretically possible when examining deep chest muscles with pleural penetration; nerve/muscle injuries—although the risk is low when performed by experienced personnel, there is a minimal risk of direct injury to nerves or blood vessels.
- Relative Contraindications: anticoagulant/antiplatelet therapy—requires caution and a risk/benefit assessment; pacemakers/defibrillators—although there are no absolute contraindications, caution and communication with the attending physician are necessary; local skin infections—avoid inserting the needle into areas with active infections.

Performing and interpreting needle EMG requires a high level of expertise and specific training on the part of the neurophysiologist. The choice of muscles to be examined and the interpretation of the tracings are crucial for an accurate diagnosis. Although there are guidelines, small variations in technique can affect the results.

Because it is an invasive and timely test, it is not suitable for prolonged monitoring or for studying muscle activity during complex movements or daily activities, for which surface electromyography is more suitable.

Needle EMG is therefore a specialized test that requires specific equipment and qualified personnel, which can make it more expensive and less available than other methods. It is a powerful and indispensable diagnostic tool for the detailed assessment of neuromuscular disorders, offering a level of specificity and precision unattainable with other methods. The need for specific expertise limits its use to cases where it is strictly indicated for a thorough diagnosis. Furthermore, it is an invasive method that can cause pain and discomfort to the patient [59].

## 2. Materials and Methods

This clinical-observational study was performed between July 2024 and February 2025 at the School of Dentistry of the University of Bari, Italy, in accordance with the provisions of the Helsinki declaration and approved by the Research Ethics Committee (REC) of Polyclinic of Bari (No. 4900/93348/2015). Ethical approval and informed consent from each human subject were obtained. Twenty patients (10 men, 10 women) were enrolled in the study group.

- Inclusion criteria were:
  - age between 18 and 30 years old;
  - full dental arches (from 7 to 7);
  - Caucasian ethnic group.
- Exclusion criteria were:
  - partially or totally edentulous dental arches;
  - previous facial trauma,
  - maxillofacial surgery.

The average age of the patients was 24.8 years old and the standard deviation was 1.29.

The TMDs were assessed following the standardized Diagnostic Criteria for Temporomandibular Disorders (DC/TMD). A single experienced practitioner valued symptoms and signs through an anamnestic questionnaire and clinical examination.

### 2.1. Patient’s History

The operator must record the general physiological, pharmacological and remote pathological anamnestic history up to the specialist remote and immediate anamnestic history.

### 2.2. Clinical Examination

**Figure 1:**
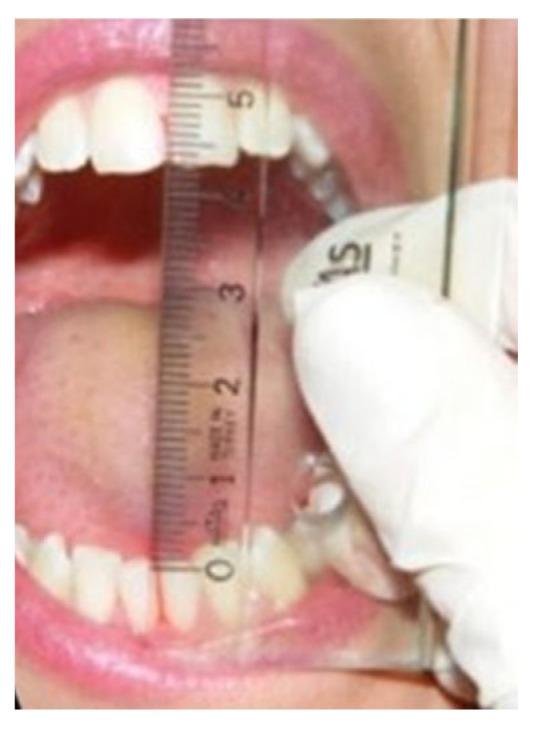
Endfeel measurement.

Endfeel: this parameter assesses the quality of movement perceived by the examiner at the end of the passive range-of-motion and is conditioned by the structures that are limiting the movement. It is tested by placing the thumb and the index fingers between a patient’s upper and lower incisors at the maximal active mouth opening and applying a downward force to passively increase the incisal distance (Figure 1). A soft endfeel and increased mouth opening suggests muscle-caused restriction, while a hard and not increasable opening is more likely to be associated with intracapsular sources (e.g., disc displacement without reduction).

Both the maximal active and the passive openings were measured using a caliper or ruler. Endfeel was recorded as “positive” when it was greater than 2 mm., i.e., the physiological stretching of the ligaments (joint play)—allowing the evaluation and comparison of the extent of a paucisymptomatic muscle contracture not as a subjective complaint, but in numerical terms.

Finally, TMJ diagnostics consists of specific first-level instrumental tests, such as orthopantomography (OPT). Level II diagnostics are imaging tests that include MRI and CT.

#### 2.2.1. Morphological Occlusion

The type of occlusion was assessed bilaterally, according to the angle classification, at the level of the canine and first molar.

The diagnosis of posterior crossbite at the level of premolars and molars was made in cases of occlusion in which the supporting cusps of the upper teeth are functionally cutting cusps.

Anterior open bite was diagnosed in cases of overbite less than or equal to 0 mm and in the absence of incisal contact.

Deep bite was diagnosed in cases of overbite greater than or equal to 5 mm.

Our sample was made up of the following:

- 1 open bite;
- 1 posterior cross-bite;
- 3 deep-bites.

#### 2.2.2. Functional Occlusion

Using a millimeter ruler or a caliper, maximum active opening (range of voluntary opening accomplished by the patient) and passive opening (obtained by slight forcing exerted by the operator) was assessed. This measurement allows quantification of the endfeel, i.e., the difference between passive and active opening, a clinically relevant parameter for identifying any functional limitations or neuromuscular interference in mandibular movement.

Two-colour (red–blue) occlusal paper of 80 μm thickness was used to record the occlusal contacts in centric occlusion and in the mandibular movements of protrusion and laterality. Each patient was positioned so that the Frankfurt plane was parallel to the floor, and then then asked to bite the occlusal paper and evaluate the occlusal contacts in centric occlusion and in the mandibular movements of protrusion and right and left laterotrusion. The possible presence of precontacts and/or interferences was reported in the gnathological record of each patient, specifying, where necessary, the number of teeth involved in the occlusal relationship.

### 2.3. Teethan^®^

Teethan is a device that allows a surface electromyographic analysis of the main masticatory muscles and a functional analysis of dental occlusion in order to quantify the influence of the occlusal state on the patient’s neuromuscular balance.

#### 2.3.1. Advantages of Teethan Method

Teethan offers a range of benefits that make it a valuable addition to dental practice. Unlike purely clinical assessments, which can be subjective, Teethan provides precise numerical and graphical data on muscle activity. This allows for the quantification of occlusion balance or imbalance. The results are recorded and documented, allowing for objective monitoring of treatment progress over time and providing important clinical documentation.

It helps identify asymmetries in muscle activity or muscle hyperactivity, which may be indicators of latent or symptomatic malocclusions or temporomandibular disorders (TMDs). Objective data guides the dentist in planning more targeted and effective treatments, whether in orthodontics, prosthetics, conservative dentistry or gnathology.

It allows for the verification of the effectiveness of interventions (e.g., the application of a bite guard, the optimization of a prosthesis or the completion of orthodontic treatment) by measuring the improvement in muscle balance [60].

#### 2.3.2. Limits of Teethan Method

The graphs and indices provided by Teethan are easily understandable, even for non-specialists. This facilitates the explanation of the patient’s condition and the need for the proposed treatments, increasing compliance.

The patient can “see” their imbalance, making them more engaged and motivated in the therapeutic process. Furthermore, the exam lasts just a few minutes (3 to 5 min), making it easily integrated into clinical routines, even during a normal visit. The wireless probes are easy to place on the skin over the masticatory muscles, requiring no complex preparation. The lack of discomfort or invasiveness contributes to greater patient acceptance. Being a surface exam, it is completely non-invasive and painless. There are no significant contraindications, making it suitable for a wide range of patients, including children, pregnant women and patients with medical devices.

The majority of our sample refused to apply needles due to pain and fear, and it was precisely this last factor that led to the exclusion of needle EMG and the preference for EMGs.

Electromyographic data are not self-explanatory. Their interpretation requires a solid understanding of functional anatomy and the physiology of masticatory muscles and occlusion, as well as good clinical experience. Teethan is a support tool, not a substitute for clinical judgment and a thorough physical examination.

Electromyographic activity can be influenced by numerous non-occlusal factors, such as stress, anxiety, muscle fatigue, pain, medication intake, inflammation or even environmental temperature. These factors can alter the data and mask the true occlusal condition.

Proper execution of the test, including head position and bite stability, is crucial to obtaining reliable data. Teethan detects the presence of muscle imbalances or dysfunctions, but cannot determine the etiological cause of such problems. For example, it cannot distinguish whether an imbalance is due to a skeletal malocclusion, a joint problem (TMJ), trauma or a parafunctional habit (bruxism, clenching).

For a complete diagnosis, other investigations are often necessary, such as radiographic examinations (orthopantomography, cone beam CT), TMJ MRI, model analysis, muscle palpation, etc. Teethan assesses muscle activity in maximum intercuspation (static position). It does not provide direct information on occlusal dynamics, mandibular movements or interference during chewing or eccentric movements. Although useful for screening, it may not be sufficient to diagnose or manage more complex forms of temporomandibular dysfunction involving severe joint or structural issues.

If the patient does not follow instructions or makes involuntary movements during the recording, the data may be compromised and not representative of the actual situation.

Therefore, well aware of the limitations and factors that can influence electrode placement, we asked patients to arrive for their appointments without a beard or makeup, and we cleansed their skin with alcohol before applying the electrodes. To address the variability of electrode application points, we relied on a single experienced operator for all 20 patients, who followed a standardized method recommended by the manufacturer for electrode application. This method therefore appears to be equally valid and scientifically validated.

#### 2.3.3. Teethan Protocol

The work protocol is based on maximum clenching tests (maximum voluntary contraction) lasting five seconds each. The result obtained expresses the degree of balance of muscular activity through recognized and validated percentage indices.

The Teethan feature is the normalization of the electromyographic signal given by the ratio between two successive clenching tests, performed with and without the interposition of cotton rolls between the dental arches. This method is registered under the name Syncromyography. This guarantees the objectivity and repeatability of the measurement, eliminating all alterations that could influence the single measurement. In fact, the physiological and anatomical variability of the patient is eliminated, as well as the variability introduced by external factors, such as the incorrect positioning of the electrodes, the conductivity of the skin, interference, etc.

All acquired results are automatically archived and can be exported in PDF format, which becomes an effective means of communication with the patient, as well as with other professionals, experts and collaborators in the field of occlusal relations.

The Teethan system includes the following testing protocols:

- OCCLUSAL-STATIC TEST: protocol for measuring the balance of dental occlusion.
- DYNAMIC CHEWING TEST: protocol for the evaluation of neuromuscular coordination during the chewing act.
- OCCLUSAL TEST.

In order to measure the balance of dental occlusion, the patient must perform two clamps of five seconds each, the first with salivary rollers, the second without. The test ends after the two windows with the generation of the report.

To minimize any interference due to the patient’s posture, the back of the chair must be ensured in a vertical position, and the subject has to be placed in a relaxed position with legs uncrossed and hands resting on the knees, and looking forward.

Each muscle is identified by a characteristic colour. During the execution, the electrical activity of the analyzed muscles is displayed in real time, with the use of bars, which become more coloured the stronger is the activation of the muscle associated with them. Recording stops automatically after five seconds and goes directly to the next acquisition phase. It is possible, however, to carry out and compare multiple calibration tests to select the one deemed best. The software is ready to carry out the actual occlusal test. It is made up of the following sections:

##### Parameters and Indices of the Occlusal Plane

**Figure 2:**
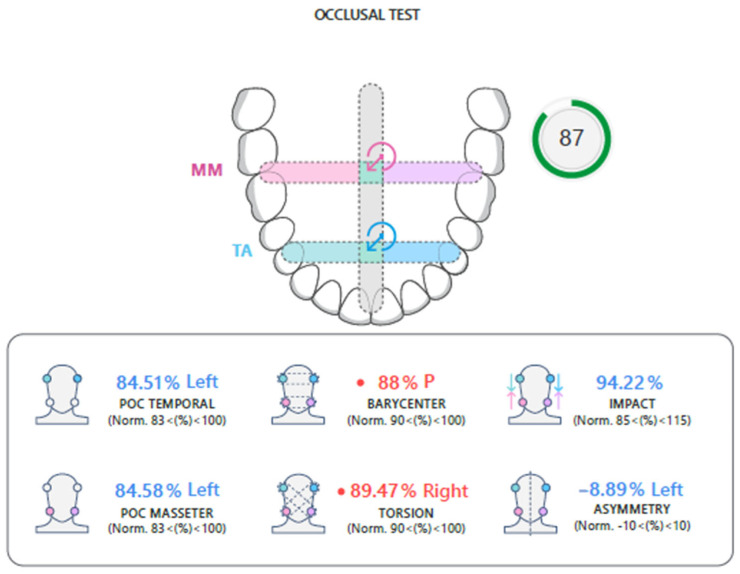
Occlusal test report. MM: Masseter Muscle. TA: Anterior Temporal.

The representation of the dental arch contains two targets (Figure 2), whose position summarizes the occlusal condition, according to the calculated indices:

- The blue target refers to the activity of the temporalis muscles (which govern the front part of the mouth);
- The pink target refers to the activity of the masseter muscles (which govern the back of the mouth).

The dotted bands represent the normality bands of the reference indices. The intersections of the bands represent the areas in which both targets fall in case of balanced occlusion.

The Global Neuromuscular Balance Index is displayed immediately next to the arch figure.

Green: when the global balance is greater than 83%.

Yellow: when the global balance is between 82% and 75%.

Red: when the global balance is less than 74%.

**Figure 3:**
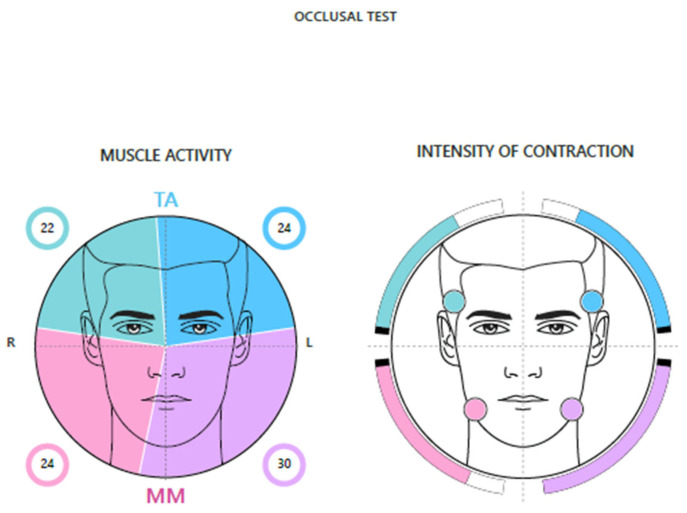
Muscle activity diagram.

- Muscle Activity and Intensity of Muscle Contraction (Figure 3).

It is possible to view the distribution of average and normalized muscle activities but also the contraction intensity of each muscle investigated. In the first case, each sector of the pie diagram is more colourful the more dominant the activation of that muscle is.

With a good neuromuscular balance, corresponding to the absence of malocclusion, the colours will be equally distributed in the four quadrants, and each sector will occupy a portion equal to 25%. In the second case, however, the muscle that expresses the greatest amplitude value is represented with a complete bar, while the remaining bars are represented proportionally to it.

##### Muscle Electrical Activity

The raw electrical activities, i.e., the signals detected by the electromyographic probes, of the masseter and temporalis muscles are shown in the aforementioned panel.

##### Normality Values and Notes

In the aforementioned panel, it is possible to view the self-reading of non-standard data and add clinical notes to customize the report. In the upper area, non-standard values are highlighted with a check. This section is filled automatically, and no changes can be made. In the lower area, however, it is possible to add notes and any information related to the occlusal test carried out. The chewing test analyzes in a “dynamic” condition neuromuscular coordination during the chewing act, while the occlusal protocol of the neuromuscular balance is evaluated in a “static” condition.

The patient is given a chewing gum and asked to chew only on the right side. The test then starts and stops automatically 15 s later. Finally, the patient is asked to move the gum to the left side and chew only on the left. The test is repeated and interrupted again after 15 s. At the end of an acquisition, the data report is automatically generated. The clinical report of the chewing test is composed of two sections:

- Chewing Report;
- Notes page.

**Figure 4:**
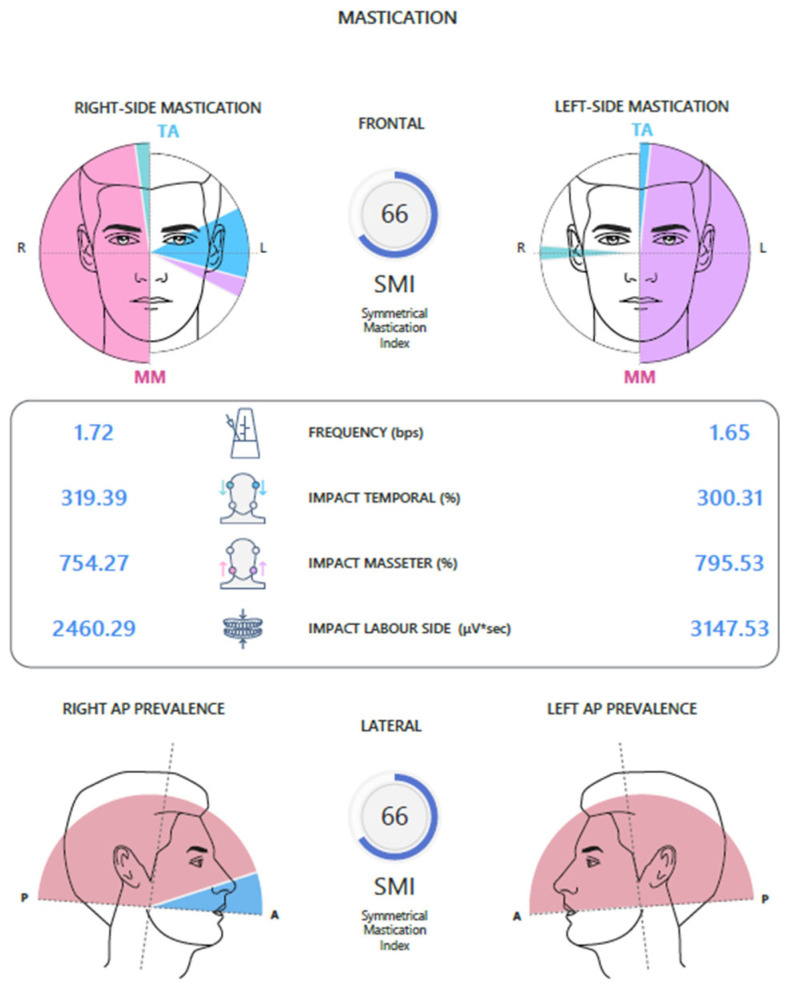
Chewing test.

On the Chewing Report screen, the test results can be viewed in two ways: frontal and lateral (Figure 4).

The frontal mode shows the right and left side chewing test with the relative activities of the masticatory muscles that contributed to the chewing act.

In the lateral mode, the antero-posterior prevalence of chewing on the right and left side is indicated, with a greater activation of the temporal muscles or masseters on the working side. In addition to graphical representation, both visualization modes provide objective quantification of neuromuscular activity by calculating specific numerical indices for each chewing side, which are useful for comparative analysis of functional symmetry and muscle efficiency during chewing. Below are the numerical indices:

Frequency: number of chewing acts per second;

Temporal Impact (%): index of the muscular work performed by the anterior temporalis (TA) during the test;

Masseter Impact (%): index of the muscular work performed by the masseter muscles (MM) during the test;

Working Side Impact: muscular work of the right side (right temporalis and right masseter) and of the left side (left temporalis and left masseter), not normalized.

In the Notes Page, you can add any notes you want to insert into the chewing test.

In the case of a patient wearing glasses, the test was performed both with and without glasses.

**Figure 5:**
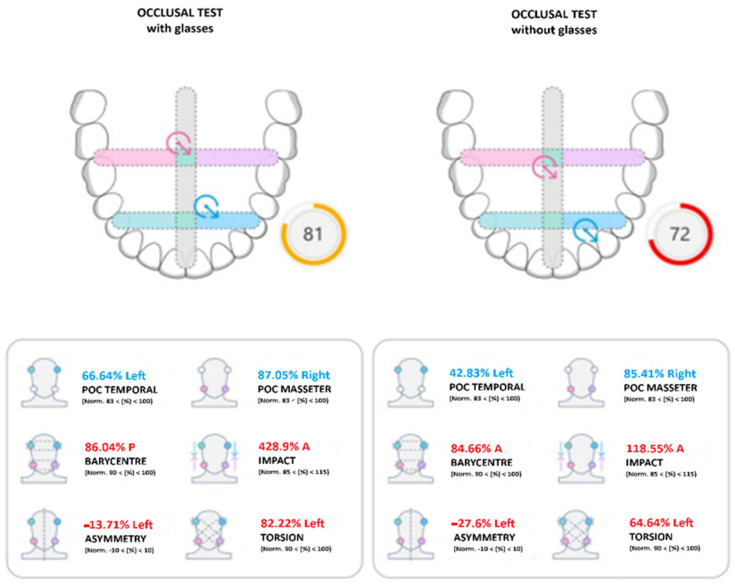
Indices obtained with the occlusal test.

The occlusal test allows to obtain the following 5 indices and the relative normality band (Figure 5).

- Percentage Overlap Coefficient (POC): refers to the overall activity of the temporal and masseter muscles, specifying which muscle of each pair prevails.
- Normal values are between 83% and 100%.
- Center of gravity (BAR): compares the activity of the temporal muscles and the masseter muscles; physiologically, a posterior center of gravity is preferable, that is, with prevalent activity of the masseters compared to the temporals.
- Normal values are between 90% and 100%.
- Torsion (TORS): evaluates the crossed activity of the temporal muscles and the masseter muscles, revealing the torsion of the mandible on the horizontal plane.
- Normal values are between 90% and 100%.
- Muscle Work (IMPACT): refers to the intensity of the muscular work of the temporalis muscles and masseter muscles. A high intensity reveals a tightening patient, while a low intensity reveals the presence of nociceptive pain. Normal values are between 85% and 115%.
- Asymmetry (ASIM): compares the activity of the temporal and masseter muscles on the right side and the activity of the temporal and masseter muscles on the left side, allowing identification of any asymmetry and the dominant side on the occlusal plane. Normal values are between −10% and 10%.

The chewing test, whose purpose was to evaluate neuromuscular coordination in a dynamic condition, involves chewing a piece of gum for 15 s on the right side and for 15 s on the left side. In the case of a patient who wears glasses, the test was performed both with glasses and without glasses.

**Figure 6:**
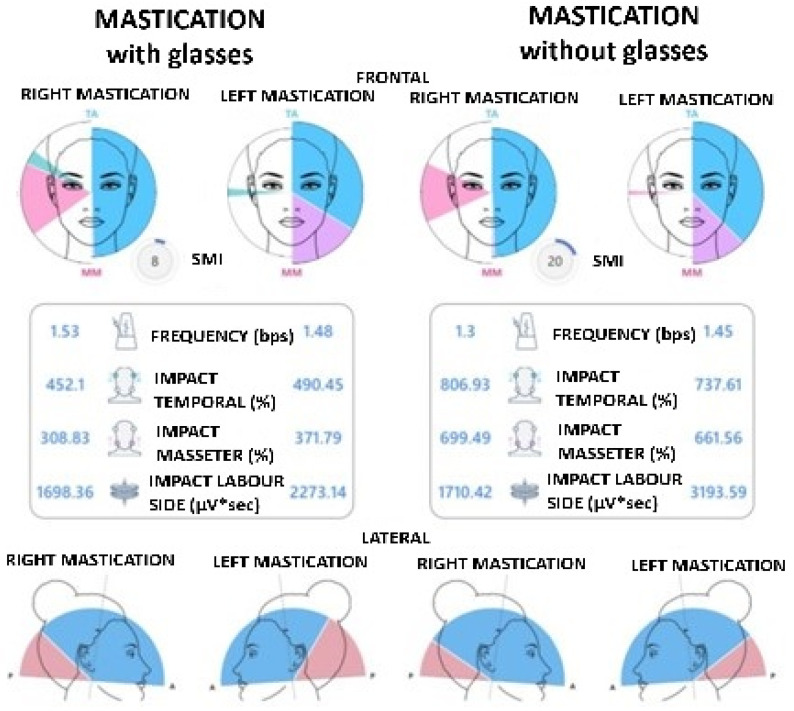
Indexes obtained with the chewing test.

The chewing test allows to obtain the following indices:

- Global Symmetry Index (SMI): compares neuromuscular coordination during right and left chewing. The optimal value is 100%.
- Chewing y: refers to the number of chewing acts per second.
- Work Produced: evaluates the work produced by the temporalis and masseter muscles during the chewing cycles performed, as well as the work of the involved side, not normalized (Figure 6).

### 2.4. Statistical Analysis

Statistical analysis was performed using the software “Prism” (1994–2024 GraphPad Software, LLC, version 10.4.1 (532), 3 December 2024, San Diego, CA, USA). Data comparison was performed using Student’s *t*-test.

## 3. Results

Data analysis was conducted using descriptive statistics tools, integrated with the use of the Student’s *t*-test to verify differences between independent samples. This test, belonging to the parametric test category, allows evaluating whether the differences detected between the means of two groups are statistically significant or attributable to the random variability intrinsic to the data. The significance threshold was set at *p* < 0.05, a value commonly accepted in biomedical research as indicative of differences not attributable to chance.

In particular, the *t*-test was applied to the comparison between the electromyographic results obtained from two subgroups of subjects: one who performed the tests wearing glasses and one without vision correction. The comparison involved both the measurements taken during the static clenching test in maximum intercuspation and those acquired during the chewing activity. The analysis revealed significant differences in some parameters, suggesting a potential role of the visual component in influencing the neuromuscular recruitment of the masticatory system.

### 3.1. Occlusal Test Results (Static Condition)

**Figure 7:**
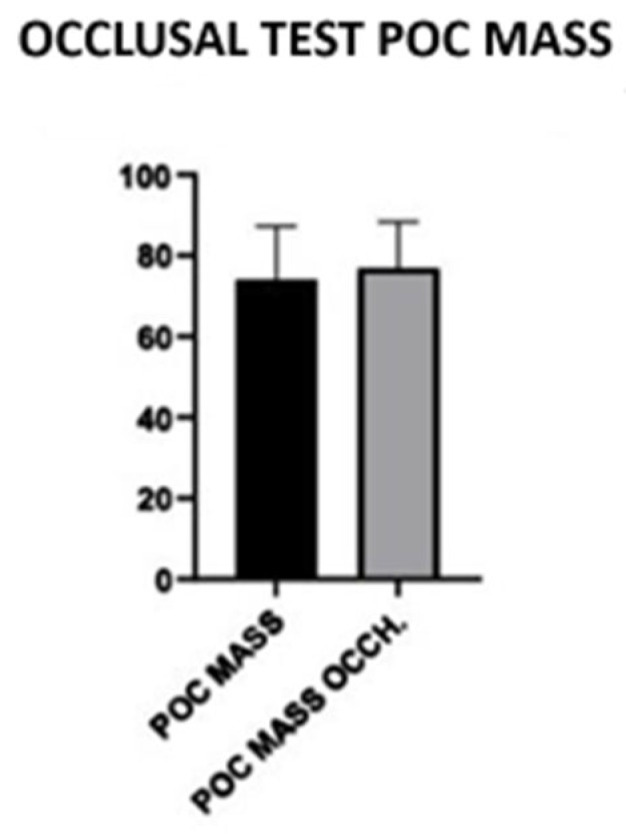
Histogram of POC MASS with or without glasses.

In the maximum intercuspidation (MI) clenching test, five main electromyographic parameters were analyzed: POC (Percentage Overlapping Coefficient), BAR (Center of Gravity), TORS (Torsion), IMPACT (Muscle Work) and ASIM (Muscle Asymmetry). Among these, the POC MASS parameter showed a statistically significant difference between subjects wearing glasses and non-wearing subjects (Figure 7). In particular, the group that performed the test wearing glasses showed a mean POC MASS value of 76.8%, compared to the value of 71.74% in the group without glasses. This difference, associated with a *p*-value of 0.0042, suggests a possible influence of visual balance on the symmetric activity of the masseter muscles, confirming the functional interconnection between the visual system and orofacial neuromusculature (Table 1).

Another significant parameter was IMPACT, an indicator of the overall muscle load during clenching. Also in this case, an increase was observed in subjects with glasses (mean: 176.2) compared to those without (mean: 103.5), with *p* = 0.0429. This data suggests that ocular balance can contribute to better muscle recruitment and greater occlusal efficiency, supporting the hypothesis that visual proprioceptive stimuli can modulate man. 

### 3.2. Dynamic Test Results (Chewing)

The second protocol, focused on the dynamic analysis of masticatory function by chewing gum, involved the recording of indices relating to frequency, symmetry, muscle work and lateral dominance. The masticatory frequency index, expressed in chewing acts per second, showed a significant prevalence on the left side compared to the right side, with a higher mean value and statistical significance (*p* = 0.0131).

This finding was further supported by the analysis of electromyographic graphic reports, which showed greater muscle activation on the left side during chewing.

The observation of such functional asymmetry could be interpreted as an adaptive response to latent occlusal imbalances or consolidated chewing habits. Furthermore, it has been noted that, in subjects with left-sided chewing prevalence, the number of occlusal contacts also tends to increase on the same side, suggesting a correlation between neuromuscular activity and occlusal stimulation.

The Pearson R correlation test was used to correlate occlusal parameters and quantitative clinical data regarding opening. This test reveals a possible linear relationship between two statistical variables. It has a value between −1 and +1: +1 expresses a perfect positive linear correlation, 0 expresses an absence of linear correlation, while −1 indicates a perfect negative linear correlation.

**Figure 8:**
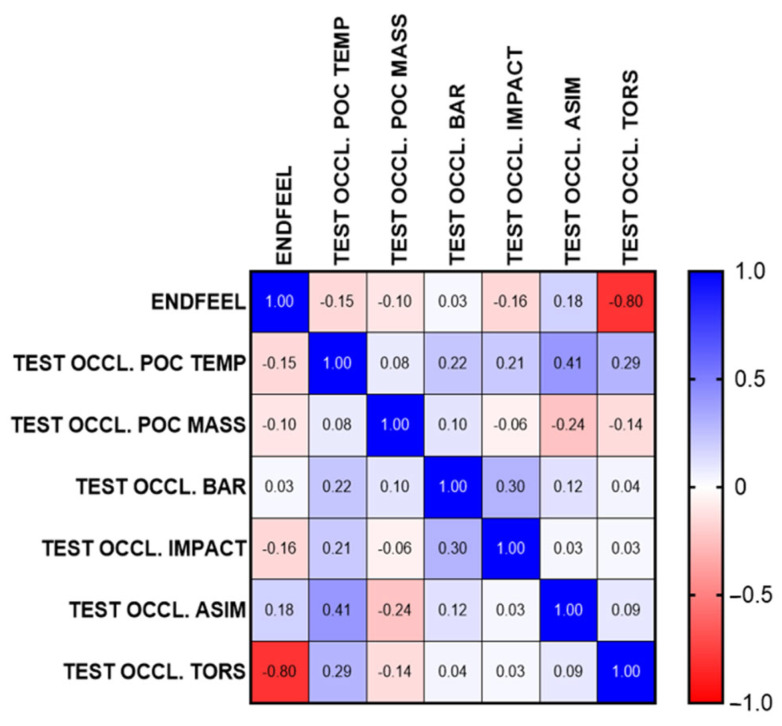
Correlation matrix which shows the correlation indices between two or more variables.

The correlation matrix is a square table that reports the correlation indices between two or more variables (Figure 8).

Further evidence of correlation is provided by performing the clinical endfeel maneuver with subsequent evaluation of the relationship between the value of passive opening and the occlusal parameter of mandibular torsion.

### 3.3. Correlations Between Clinical Variables and EMG Parameters

The correlation analysis between occlusal variables and clinical parameters highlighted an inversely proportional relationship between the value of mandibular torsion (TORS) and the measured endfeel. The Pearson coefficient indicated a negative correlation (r = −0.80), suggesting that as the endfeel increases, there is a reduction in torsion, often below physiological limits (90–100%). In particular, a high endfeel, indicative of an increase in resistance in passive mandibular movements, was associated with a reduction in torsional symmetry, potentially indicative of occlusal interferences or functionally significant precontacts.

This link was also confirmed by the collected clinical data, where patients with endfeel greater than 4 mm showed TORS values below the physiological threshold, while subjects with endfeel between 1–2 mm presented mandibular torsion within normal limits.

### 3.4. Muscle Symmetry Analysis

Another particularly relevant aspect that emerged from the electromyographic analysis concerned the muscular asymmetry between the right and left sides of the masticatory muscles. The ASIM parameter, which represents the degree of global balance between the two antagonistic muscular components, showed lower values, on average, in subjects with mild occlusal alterations, suggesting the presence of an unbalanced distribution of muscular strength. This condition, although asymptomatic in some subjects, could represent an early signal of a neuromuscular imbalance, potentially evolving towards a dysfunctional condition.

In subjects with manifest occlusal asymmetries (midline deviation, unilateral or bilateral cross-bite, premature contacts), the ASIM index recorded higher values, reflecting a lower neuromuscular stability during clenching. This observation is consistent with the concept of proprioceptive feedback disturbed by occlusal anomalies that, over time, can induce a modification of the basal muscular patterns.

### 3.5. Distribution of the Muscle Center of Gravity

The BAR (Center of Gravity) parameter provided additional information on the anteroposterior balance between the temporalis and masseter muscles. The physiological distribution should be around an ideal value of 90–100%, indicating an optimal balance between the anterior (temporal) and posterior (masseter) muscles. However, a fair amount of interindividual variability was found in the sample analyzed, with values ranging from 78% to 105%.

In particular, in subjects with active parafunctional habits (reported clenching or nocturnal bruxism), the center of gravity often shifted anteriorly, highlighting a predominance of temporalis activity over masseter activity. This alteration could be interpreted as a sign of compensatory muscular hyperactivity, potentially related to conditions of functional overload of the temporomandibular joints.

### 3.6. Chewing Frequency and Lateral Preference

Regarding the results of the dynamic chewing test, the analysis allowed the detection of interesting data related to the rhythm and laterality of the chewing cycle. Subjects with a left-handed chewing preference showed a chewing frequency that was, on average, more regular and symmetrical, with a balanced alternating activation of the bilateral masseter muscles. On the contrary, in right-handed subjects, a greater variability in the rhythm was observed, with moments of asynchronous muscle activation and a lower cyclic coherence.

These results suggest that lateral preference, often linked to postural factors or previous habits, can influence the organization of neuromuscular recruitment during masticatory function. This influence may assume clinical relevance in the planning of orthodontic or gnathological treatments aimed at rebalancing the functional occlusal load.

### 3.7. Subjective Results and Clinical Observations

During the instrumental tests, patients were given a short qualitative questionnaire, aimed at evaluating the subjective perception of the chewing act before and after the test. Some subjects, even in the absence of objective painful or dysfunctional symptoms, reported a sensation of early muscle fatigue or “discomfort” during voluntary clenching. These reports were more frequent in patients with lower-than-average POC values and with an overall reduced IMPACT, supporting the hypothesis that inefficient muscle activity can also be perceived subjectively.

Finally, in several subjects, it was possible to visually observe an improvement in muscle symmetry in the repeated test after mandibular repositioning guided by cotton rolls. This confirms the value of the normalized dynamic test in the predictive assessment of the potential for neuromuscular reharmonization through targeted therapeutic interventions.

## 4. Discussion

The analysis of the data obtained highlighted significant relationships between occlusal function and the use of visual aids, especially glasses, as well as a correlation between electromyographic parameters and mandibular mobility [58,61,62,63]. These results, although obtained on a limited clinical sample, highlight the importance of integration between postural, neuromuscular and sensorial systems in the stomatognathic function [64,65,66,67].

In the occlusal test, the POC MASS (Percentage Masseter Overlap Coefficient) was higher in subjects wearing glasses than in non-wearers [68,69,70,71]. This data, although with a slight difference, suggests that a correct visual balance can have a positive impact on the balance of mandibular muscle activity [72,73,74,75,76,77]. The visual system contributes to global postural control, and variations in visual stimulation can influence the distribution of muscle activity at the cranio–cervical–mandibular level [78,79,80].

The IMPACT parameter, indicative of bite force and intensity of muscle activity, also showed significantly higher values in subjects wearing glasses (176.2 versus 103.5), suggesting greater muscle activation during moments of maximum clenching [60,81,82,83,84]. This data could indicate a tendency to greater muscle recruitment in subjects wearing glasses, perhaps due to better spatial orientation or more stable activity of the postural tonic system [85,86,87,88].

In the chewing test, a significant asymmetry in the frequency of chewing acts was highlighted, with a prevalence for the left [89,90,91,92,93,94]. This finding was clinically accompanied by an increase in occlusal contacts during functional movements, suggesting a preferential lateralization of the chewing function, which may reflect functional imbalances or neuromuscular adaptations [95,96,97,98]. The presence of a lateral chewing preference is a frequently observed element in the general population but may represent a predisposing factor to muscular or joint dysfunctions if associated with occlusal or postural alterations [99,100,101,102,103,104].

Of particular interest is the negative correlation observed between the electromyographic parameter TORS (mandibular torsion) and the endfeel value, indicative of passive mandibular mobility [105,106,107,108,109]. This correlation (value = −0.80) suggests that greater resistance to passive mandibular movement is associated with less symmetry in the distribution of muscular forces during masticatory activity [110,111,112,113,114,115]. In physiological conditions, a TORS value between 90% and 100% represents a balanced distribution of forces; its reduction is instead indicative of a functional asymmetry potentially induced by occlusal interferences or adaptive muscular patterns [41,49,116,117,118]. The increased endfeel value in subjects with altered TORS clinically confirms a functional limitation of movement, probably secondary to abnormal muscular tensions or inefficient neuromuscular coordination [119,120,121,122,123,124,125].

These results are confirmed by the biomechanical concept according to which the interaction between the sensory systems (visual, proprioceptive and vestibular) significantly influences the masticatory function and the mandibular posture [126,127,128,129]. The importance of sensory integration has already been widely discussed in the gnathological and postural literature, where it is highlighted how even small visual alterations (for example, linked to the presence or absence of glasses) can be reflected on aspects of the stomatognathic function, such as muscle tone, masticatory symmetry and bite force [130,131,132,133,134]. The Teethan device, used in this study for electromyographic recordings, represents a significant advance in the evaluation of occlusal and masticatory dynamics [25,28,135,136]. Through sensors applied to the masticatory muscles, Teethan is able to accurately measure muscle parameters, such as the intensity of muscle activity (IMPACT) and the distribution of activity between the masseters and temporalis muscles [42,44,137]. This tool also allows the detection of any muscle dysfunctions and asymmetries, proving essential in the diagnosis of temporomandibular disorders and in the analysis of occlusal interferences [138,139,140,141]. Furthermore, thanks to its ability to monitor occlusal balance and mandibular movements in real time, Teethan represents a valid support in the planning of orthodontic and gnathological treatments, allowing a more precise and personalized approach [48,142,143,144,145].

From a clinical point of view, the results of this study underline the need for a multidisciplinary evaluation of the patient, particularly in subjects who present symptoms of temporomandibular dysfunction or occlusal imbalances [34,146,147,148]. Electromyography confirms itself as a useful tool to highlight dysfunctional patterns that may not be immediately detectable with clinical examination alone [38,149,150,151].

However, it is important to consider some limitations of the study. The sample size is small and composed of young and healthy subjects, which limits the generalizability of the results. Furthermore, the lack of follow-up over time does not allow evaluation of whether the observed variations have a stable clinical impact or are transitory. Future studies with larger and more heterogeneous samples, possibly integrated with postural instrumental assessments, could further clarify the role of the visual system and neuromuscular asymmetries in mandibular function [152,153,154,155].

In light of the working hypothesis—which postulated a potential correlation between occlusal asymmetries and neuromuscular imbalance—the data obtained provide supporting evidence. In particular, the inverse correlation between mandibular torsion (TORS) and the endfeel parameter, and the modulation of masticatory muscle activity by visual input, confirm the initial clinical assumptions of a multifactorial influence on craniomandibular dynamics [156,157,158,159]. These results are consistent with previous studies highlighting the role of visual and postural factors in occlusal function and temporomandibular disorders [160,161,162,163,164].

Moreover, the observed increase in occlusal contacts on the preferred chewing side, and the asymmetries detected via EMG analysis, align with earlier findings suggesting that chronic lateralized function may lead to adaptive neuromuscular patterns [165,166,167,168,169]. This confirms the importance of including dynamic functional tests in the clinical evaluation of CMD patients [170,171,172,173,174].

From a broader perspective, these findings underscore the need to consider neuromuscular, postural and sensory influences in the diagnostic workflow [175,176,177,178,179]. Future research should aim to expand the clinical sample to include different age groups and patients with postural or ophthalmological conditions, and to assess the long-term impact of therapeutic interventions such as vision correction, occlusal splints or neuromuscular re-education on EMG symmetry and mandibular function.

## 5. Conclusions

The results of this study highlight a significant relationship between masticatory muscle activity and adequate ocular balance. In particular, the correct ocular muscle balance contributes to reducing muscle fatigue and improving the efficiency of the masticatory muscles during the occlusal test. This observation suggests that the interaction between the visual system and masticatory muscle function may play a crucial role in optimizing stomatognathic function.

Regarding the chewing test, experimental data confirm the existence of a preferred side of chewing in each individual. Clinical results also indicate that the increase in activity of the muscular side involved in the chewing act is associated with an increase in occlusal contacts. This phenomenon demonstrates how the asymmetric activation of the masticatory muscles directly influences the distribution of occlusal contacts during chewing.

Finally, the inverse proportionality relationship found between mandibular torsion and the endfeel parameter confirms the presence of a significant correlation between occlusal parameters and craniomandibular dysfunctions. The electromyographic data obtained through the Teethan device support the hypothesis that qualitative and quantitative alterations in occlusion can be predictive indicators of muscular and joint disorders, particularly affecting the temporomandibular joints (TMJs). The Teethan device provided a detailed analysis of occlusal conditions, which proved to be crucial for the early diagnosis and management of such disorders.

Unfortunately, the limitation of our study is related to the small sample size. This is likely why we were unable to detect a correlation with gender and the presence of malocclusion. We conducted the study only on healthy subjects, without symptoms of DCM and bruxism.

The neuromuscular responses to visual input may be worth exploring in future work. The goal is to further investigate this, expanding the sample to obtain significant results. Ours is intended to be a pilot study that adds a small scientific contribution. 

## 6. Patents

The authors declare that there are no patents resulting from the work reported in this manuscript.

## Figures and Tables

**Table 1:** Student’s *t*-test.

Table Analyzed	Occlusal Test POC MASS			
Column B	POC MASS OCCH.			
VS.	VS.			
Column A	POC MASS			
Paired *t* test				
*p* value	0.0042			
*p* value summary	**			
Significantly different (*p* < 0.05)?	Yes			
One- or two-tailed *p* value?	Two-tailed			
t, df	t = 3.810, df = 9			
Number of pairs	10			
How big is the difference?				
Mean of differences (B-A)	5.064			
SD of differences	4.203			
SEM of differences	1.329			
95% confidence interval	2.057 to 8.071			
R squared (partial eta squared)	0.6173			
How effective was the pairing?				
Correlation coefficient *(r)*	0.9501			
*p* value (one tailed)	<0.0001			
*p* value summary	****			
Was the pairing significantly effective?	Yes			
Normality of Residuals				
Test name	Statistics	*p* value	Passed normality test	*p* value summary
Anderson–Darling (A2 *)	0.6024	0.0844	Yes	ns
D’Agostino–Pearson omnibus (K2)	6.806	0.0333	No	*
Shapiro–Wilk (W)	0.8501	0.0583	Yes	ns
Kolmogorov–Smirnov (distance)	0.2222	>0.1000	Yes	ns
	POC MASS	POC MASS OCCH.	POC MASS OCCH.—POC MASS	
Number of values	10	10	10	
Minimum	49.57	51.53	1.03	
25% Percentile	61.77	69.05	1.843	
Median	75.56	80.78	3.835	
75% Percentile	82.37	85.41	8.018	
Maximum	85.41	87.05	14.69	
Mean	71.74	76.8	5.064	
Std. Deviation	13.12	11.52	4.203	
Std. Error of Mean	4.15	3.642	1.329	
Lower 95% CI	62.35	68.56	2.057	
Upper 95% CI	81.12	85.04	8.071	

*, **, **** and ns indicate that no value can be reported.

## Data Availability

The data presented in this study are available on request from the corresponding author. The data are not publicly available due to privacy and ethical restrictions. All patients enrolled in this study have signed the informed consent, authorizing in detail the publication of photographs, radiographs and Teethan electromyographic tracing, as the attached “draft informed consent”.

## Abbreviations

**Abbreviation**	**Definition**
AAOP	American Academy of Orofacial Pain
ASIM	Asymmetry
BAR	Center of Gravity
DC/TMD	Diagnostic Criteria for Temporomandibular Disorders
EMG	Electromyography
ENG	Electroneurography
IHS	International Headache Society
IMPACT	Muscle Work
MASS	Masseter
MI	Maximum Intercuspidation
MM	Masseter Muscle
OD	Oral Stage Dysphagia
OPT	Orthopantomography
POC	Percentage Overlapping Coefficient
RCD	Research Diagnostic Criteria
sEMG	Surface Electromyography
SMI	Global Symmetry Index
TA	Anterior Temporal
TENS	Transcutaneous Neural Electrical Stimulation
TMJ	Temporomandibular Joint
TORS	Torsion
